# Natural Products as Regulators against Matrix Metalloproteinases for the Treatment of Cancer

**DOI:** 10.3390/biomedicines12040794

**Published:** 2024-04-03

**Authors:** Md. Towhedul Islam, Nak Han Jang, Hyuck Jin Lee

**Affiliations:** 1 Department of Chemistry, Faculty of Science, Mawlana Bhashani Science and Technology University, Santosh, Tangail 1902, Bangladesh; 2 Department of Chemistry Education, Kongju National University, Gongju 32588, Chungcheongnam-do, Republic of Korea

**Keywords:** cancer, matrix metalloproteinase (MMP), extracellular matrix (ECM), natural products, flavonoids

## Abstract

Cancers are currently the major cause of mortality in the world. According to previous studies, matrix metalloproteinases (MMPs) have an impact on tumor cell proliferation, which could lead to the onset and progression of cancers. Therefore, regulating the expression and activity of MMPs, especially MMP-2 and MMP-9, could be a promising strategy to reduce the risk of cancers. Various studies have tried to investigate and understand the pathophysiology of cancers to suggest potent treatments. In this review, we summarize how natural products from marine organisms and plants, as regulators of MMP-2 and MMP-9 expression and enzymatic activity, can operate as potent anticancer agents.

## 1. Introduction

**Figure 1:**
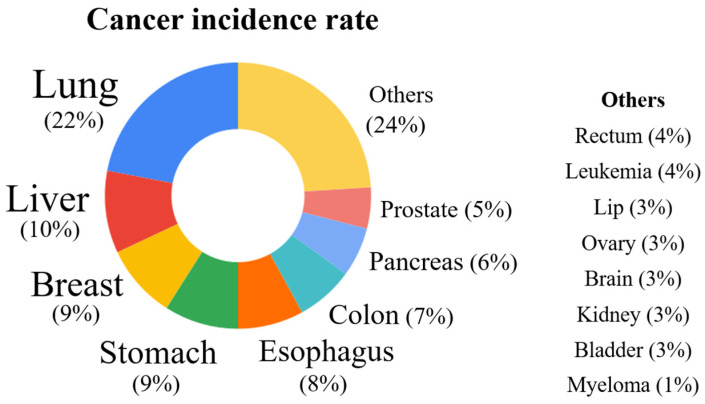
A schematic representation of the number of different cancer deaths globally.

Cancers represent a danger to human health, and they are the second leading cause of death worldwide [1]. Currently, people living in poor countries bear the brunt of sickness; a recent survey revealed that about 10 million cancer-related deaths and 19.3 million new cases were reported globally in 2020 [1,2]. The leading cause of cancer-related deaths worldwide was lung cancer (1.79 million) followed by other cancers, including liver cancer (0.83 million), stomach cancer (0.77 million), breast cancer (0.68 million), and other cancers (Figure 1) [3]. 

According to research on 29 cancer diseases across 204 nations, the overall cost of cancer to the world economy between 2020 and 2050 will be USD 25.2 trillion. About half of those will be attributed to five different forms of cancer: lung, liver, breast, colon, and leukemia [4]. Some of the key participants in the growth and aggressiveness of cancer cells of glycolytic enzymes could be lactate dehydrogenase (LDH), caspases, cyclin-dependent kinases, redox–detox enzymes, matrix metalloproteinases-2/9 (MMP-2 and MMP-9), and NAD+-dependent enzymes [5,6]. 

When a family member has a greater incidence of a certain type of cancer, it has been feasible to determine whether the disease runs in the family. The most common hereditary cancer type is colorectal cancer, by mutation of DNA MMR genes. In addition, breast and ovarian cancer are due to mutations of the BRCA1 as well as BRCA2 genes, respectively. However, 20–30% of instances of breast cancer, which affects 10 in 100 women, are genetically predisposed to BRCA1 and BRCA2 gene mutations [7,8]. Moreover, rapid industrialization and inappropriate pesticide usage are also responsible for lung, liver, and prostate cancer [9]. 

Particularly, it is difficult to diagnose cancer until it reaches the metastasis stage, consequently lowering longevity [10]. To cure cancer, different types of medical technology have been used including radiation, surgery, and systemic hormonal therapy. Surgery has been performed on about 56% and 18% of those in stages I and II. The majority of people (62%) in stage III receive chemotherapy to treat cancer, though expensive. As an alternative to surgery and chemotherapy, the administration of natural compounds will be used to modify the genes of cancer cells to mitigate cancerous death [11]. 

Throughout malignancy, tumor cells interact with surrounding endothelial cells as well as growth regulators, cytokines, and extracellular matrix (ECM) components [12], such as gelatin, collagen, elastin, and fibronectin. The four features of cancer (angiogenesis, invasion, migration, and metastasis) are dependent on the exterior microenvironment. MMPs are crucial because they increase the interplay between cells and the extracellular matrix by breaking down several types of cell adhesion molecules [13,14]. The ECM exists in muscle niches and consists of proteins, polysaccharides, RNA, and other materials, and it is essential for maintaining homeostasis and regulating the development of skeletal muscle [15]. Recent research by Liu et al. has demonstrated that collagen I, an important component of the extracellular matrix, stimulates focal adhesion kinase activation, which in turn controls NF-κB nuclear translocation and improves myoblast migration [16]. Thus, it could be concluded that ECM plays a critical role in maintaining the physiological function of satellite cells as well as skeletal muscle development. ECM variations are primarily caused by two mechanisms: (1) chemical modification, which alters the biochemical and structural properties of the ECM, and (2) proteolytic degradation, which releases bioactive ECM fragments and ECM-bound factors and may be essential for the release of cellular restrictions such as migratory barriers [17]. The degradation of the ECM by MMPs is a major factor of change in cell shape. For instance, when epithelial cells lose their polarity and cell–cell adhesion is disrupted, it facilitates the movement and infiltration of these cancer cells [18,19]. Hence, MMPs promote tumor growth and spread through the capillary endothelium [20]. 

The history of the therapeutic use of natural resources dates back thousands of years when medicinal plants rich in phytochemicals and microbes were an important constituent of medicines. This knowledge is frequently passed down from one generation to the next. Generally, natural bioactive materials derived from plants, minerals, animals, and microorganisms have been utilized to treat a wide range of illnesses [21]. Natural products could inhibit MMPs by interacting with or chelating out the catalytic Zn(II) of MMPs. Preclinical studies have shown that the hydroxamate-based MMP inhibitors, including Batimastat and Marimastat, have potential to cure cancer [22,23]. However, the anticipated outcomes of studies on patients were insufficient. Later, structural investigations using X-ray crystallography and computer simulations were used to create several hydroxamate derivatives, especially helping to increase their sensitivity as MMP inhibitors to limit cancer [22]. In this review, we first briefly address MMPs and their critical roles in human health and cancer progression; then, we address natural products from marine organisms and plants that could regulate MMP-2 and MMP-9 expression levels and activities. We tried to provide general knowledge for development of new drugs with the potential to control MMPs, leading to the successful treatment of cancers.

### 1.1. Basic Information on MMPs

**Figure 2:**
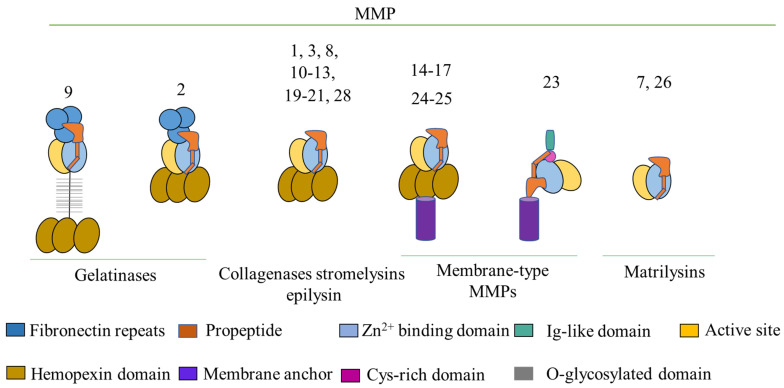
A brief description of the structure of MMPs and arrangement of their functional domains.

MMPs are Zn(II)-dependent endopeptidase [24]. MMPs have several domains including (i) membrane anchor domain and (ii) Zn(II)-binding domain, together forming the (iii) active site for substrate catalysis (Figure 2). MMPs are released by cells as (iv) proenzymes (propeptide), which are dormant and must undergo chemical adaptation by reactive oxygen species (ROS) to catalytically activated proteases [25]; (v) fibronectin repeats, which enable them to bind big substrates like collagen and effectively break gelatins [26]; and (vi) a hemopexin domain, which plays a role in ties to both substrates and inhibitors [27,28,29]. Furthermore, MMP-9 possesses a distinct (64 amino acid) linker sequence that joins the hemopexin domain, known as (vii) the O-glycosylated domain, to (viii) the active site. The most recent atypical MMP is MMP-23; it contains an immunoglobulin-like domain and cysteine-rich domain linked to the C-terminus and a transmembrane domain bound to the N-terminus [30]. Therefore, by trapping cis Kv1.3, MMP-23 affects cellular functions whereas in trans-Kv1.3, it blocks extracellular functions. Breaking the bonds in protein affects cytokine and chemokine signaling. However, MMP-23 activity in melanoma cancers can assist disease progression through these mechanisms [31]. The primary function of MMPs is to disintegrate ECM components. MMP-mediated breakage of ECM components plays a role in triggering the discharge of growth hormones from the ECM, propagation, and tissue reconstruction of cancer cells [32].

Among multiple MMPs, MMP-2 and MMP-9 have been reported to be involved in pathology of cancers. MMP-2 (gelatinase A) could break down type I, type II, and type III collagen and is produced by stroma cells in the majority of organs, including hematopoietic, endothelial, dendritic, fibroblast, mast, and macrophage cells. MMP-9 (gelatinase B) is prevalent in healthy tissues and released by dendritic, hematopoietic, macrophage, neutrophil, fibroblast, and lymphocyte cells [33,34].

**Figure 3:**
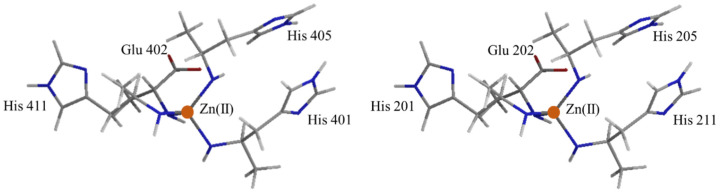
Catalytic site of MMP-2 (**left**) and MMP-9 (**right**). The N, O, C, and H atoms are presented in blue, brown, gray, and white, respectively. Zn(II) is indicated in orange circle.

The structures of MMP-2 and MMP-9 are similar, but slightly different at the catalytic center. For MMP-2, the catalytic center is composed of Zn(II) coordinated by three histidines (His201, His205, and His211) and glutamic acid (Glu202). The structure of the catalytic domain of human MMP-9 is composed of the Zn(II) coordinated by three histidine residues (His401, His405, and His411) and glutamic acid residue (Glu402) (Figure 3) [35].

### 1.2. Normal and Disease Conditions

The ECM aids in intercellular communication, signal transduction, and the regulation of biological processes including cell division and death [36]. ECM remodeling is an important and necessary process that takes place in both physiologically normal and diseased conditions. During its remodeling, cells entirely or partially destroy certain components of the ECM, such as laminin, collagen, and fibrin [37]. MMPs are among the proteases that mediate the breakdown process by inducing remodeling in both temporal and spatial dimensions [38]. Therefore, regulation of MMPs is strictly maintained to keep homeostasis. Dysregulation in any regulatory system leads to an aberrant expression of MMPs, which can exacerbate tissue degradation disorders such as fibrotic illnesses, osteoarthritis/arthritis, neurological illnesses including glaucoma, Parkinson’s disease, Alzheimer’s disease, and Japanese encephalitis, which have been linked to aberrant MMP expression [39,40]. Under normal circumstances, MMP expression is typically relatively low; nonetheless, higher MMP levels have been seen in several cancer types and are associated with increased tumor development and proliferation [41].

### 1.3. Growth of Cancer Cells through Blood

**Figure 4:**
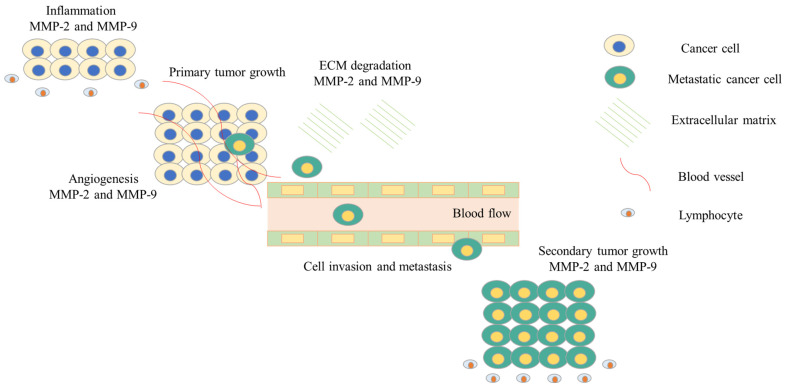
Role of MMP-2 and MMP-9 in cancer progression.

Cancer cell invasion, proliferation, and metastasis are caused by the degradation of ECM proteins [42]. Many ECM components, such as collagen, laminin, gelatin, fibronectin, elastin, and fibrin, are disrupted by MMPs and are linked to the integrity of the basement membrane. Because of this, MMPs play a critical role in the processes of cancer cell metastasis, which includes the growth of metastatic tumors, basal membrane degradation and subsequent invasion into blood or lymphatic vessels, activation of various growth factors required for angiogenesis, and extravasation into new tissues (Figure 4) [43]. Cancer cells rely on the overexpression of MMP-2 and MMP-9 to travel from the source cells to nearby tissues [43]. Furthermore, the degradation of basement membrane components such as collagen and laminin can cleave endothelial/epithelial cell barrier proteins [44]. When the tumor cells have propagated to a secondary organ, MMPs help to modify the tissue microenvironment to ensure the survival of metastatic cells. By liberating vascular endothelial growth factor (VEGF) from angiogenic islets, MMP-9 is known to initiate the angiogenic migration in cancer, [45] whereas MMP-2 controls the bioavailability of dissolved VEGF-A and tumor vascular patterning [46]. For instance, MMP-9 can cleave the interleukin-2 receptor-α (IL-2R-α) in order to inhibit the growth of lymphocytes called T cells [47]. Several previous studies claim that MMPs inhibit TGF-α activation, thereby decreasing T cells’ capacity to combat tumors [48,49]. It has also been observed that MMPs generate soluble TNF-α, which encourages cancer cells to undergo apoptosis [50]. Blocking MMPs seemed to be a viable strategy against cancer and metastatic growths. MMPs are important regulators for the progression of cancer, particularly metastasis. A number of MMP inhibitors have been practically investigated in the past decade for their potential for curbing metastasis and treating tumors. Marimastat was among of the first MMP inhibitors and underwent phase III clinical trials. The overall survival of patients with pancreatic cancer was found to be improved by Marimastat; however, the side effects of the treatments included musculoskeletal toxicity [51]. Particular MMP inhibitors are currently being developed to combat various cancers; though, blocking MMPs alone may not be enough to stop the progression of cancer.

### 1.4. Related Diseases

Some metalloproteinase-related polymorphisms and genes have an impact on the development of hypersensitivity pneumonia, an inflammatory illness brought by an overreaction of the immune system to particular organic particle inhalation [52]. Lung function decline appears to be associated with remodeling of the ECM in the airways and respiratory interstice. The variability of circulating active MMP-9 levels is examined during renal replacement therapy (RRT) for a disease that is associated with a high mortality risk and may be made worse by persistent inflammation [53]. 

Aggressive brain tumors have been revealed to have higher tissue levels of MMP-2 and MMP-9 [54,55,56,57]. Patients with malignant brain tumors also had a lower duration of survival when their tissues had positive levels of MMP-2. Cerebrospinal fluids of patients with brain malignancies have been found to contain both latent and active versions of MMP-2 and MMP-9 [58]. The activity of plasma MMP-2 and MMP-9 is considerably elevated in metastatic prostate cancer [59]. Moreover, upregulation of MMP-2 in tumor cells was linked to reduced disease-free survival. Metalloproteinase (MMP-2 and MMP-9) were found to be important signs of cancer return upon analysis of MMP-2 and MMP-9 levels in radical prostatectomy tissues [60,61]. MMP-9 plays an important role in the onset, progression, and metastasis of gastric [62], lung [63], colon [64], and breast cancers [64]. A high level of MMP-2 and MMP-9 has been found in several cancer types, such as those of the bladder, breast, bronchopulmonary, cervical, colon, glioma, larynx, lung, ovary, melanoma, myeloma, esophagus, pancreas, prostate, skin, and stomach [65].

## 2. Regulators of MMP-2 and/or MMP-9

### 2.1. Advantages of Applying Natural Products

Various natural products that are extracted from plants and marine species have potential of medicinal properties. They could be used to cure cancer and other illnesses for a long time because of their effectiveness against various impairments [66]. These compounds’ fabulous offshoots against cancer cells indicate that their oral administration is a tremendous discovery of science. Moreover, it has been revealed that almost 60% of natural products such as flavonoids, tannins, alkaloids, and terpenoids could act as anticarcinogenic agents [67,68]. They can control the growth, migration, and apoptosis of cancer cells by altering metabolic pathways as well as biological systems. Preparing anticarcinogenic medicine in a laboratory is expensive, tedious, and time-consuming, whereas the usage of natural products as drugs is relatively easy [69]. However, researchers take into consideration that natural products may be detrimental to patients due to their potent toxicity concerns [70,71]. 

### 2.2. Natural Products from Marine Organisms

Emerging research over the previous fifty years has demonstrated the positive effects of several natural compounds originating from marine life (Table 1) in both the avoidance and amelioration of cancer [72]. New bioactive agents have been abundantly produced by marine natural products [73,74,75]. The sea’s distinct climatic circumstances and variety of marine ecosystems are mostly unexplored sources of new compounds with noteworthy chemical novelty [76,77,78]. 

**Lemnalol** (Table 1), a compound that comes from soft corals (*Lemnalia cervicornis* and *Lemnalia tenuis Verseveldt*), exhibits anti-inflammatory properties on mast cells (MCs) and osteoclast function. An immunohistochemical analysis revealed that it reduces MC infiltration and degranulation, and this impact may be partially attributed to decreased MMP-9 activity [79]. **Ageladine A** is one kind of natural product derived from *Agelas Nakamura* (a marine organism). While the N-methylated derivatives failed to inhibit MMP-9, they suppressed both MMP-2 and MMP-9 proteolytic activity, based on in vitro research on isolated enzymes (Table 1) [80]. Many MMP inhibitors act by chelating out the Zn(II) from the catalytic domain [80].

Additionally, **11-Epi-sinularoide acetate** (11-epi-SA) was isolated from a soft coral *Sinularia flexibilis* (Table 1). In different concentrations, it inhibited cell migration and invasion in hepatocellular carcinoma cells (HA22T cells). MMP-2 and MMP-9 activity and expression levels significantly diminished at non-toxic dosages (7.98 M), indicating that the action was highly related to the regulation of MMPs or their inhibitors [81,82]. **Dihydroaustrasulfone alcohol** (DA) was extracted from marine coral and exhibits antioxidant as well as anticancer properties (Table 1). Moreover, numerous dosage-dependent studies reveal that it inhibits the invasion and activity of human non-small-cell lung cancer cells (NSCLC A549 cells).

Gelatin zymography analysis also showed significantly decreased levels and actions of MMP-2 and MMP-9. These results established a connection between DA’s antimetastatic effect and the suppression of colonization-related enzymes [83,84].

**Marimastat** is a major MMP inhibitor, which hinders the activity of MMP-2 and MMP-9. Regardless, the t-butyl and α-hydroxyl groups, respectively, increased solubility in water, and it was demonstrated to be oral permeable [85]. The effectiveness of this medication against models of breast and lung metastases has been shown in preclinical studies [86]. Although it has several harmful side effects, such as cell death, fibrosis, bleeding, and weight loss, it showed fabulous improvement in patients having advanced gastric cancer [86,87]. In particular, *Aplysina aerophoba* sponges produce the brominated antibiotic aeroplysinin-1, which sets off chemical defense feedback. It could exert antitumor and antiangiogenic effects. MMP-2 levels were lower in a study [88] that used multiple human endothelial cell lines (Table 1). Fluid isolates of *Aplysina aerophoba* were very effective in reducing protein and mRNA expression rates linked to MMP-2 and MMP-9 in rat astrocyte conditions [89].

### 2.3. Natural Products from Plants

Many plants contain phenolic substances. A few of these compounds have been connected to invaluable well-being and illness impacts [90]. They showed some fabulous beneficial properties including anticancer, antiviral, and cell reinforcement, as well as energizing vital properties [90]. The sources and effects of natural products on the actions and expression of MMP-2 and MMP-9 are summarized in Table 2. 

Recently, multiple studies accomplished research on the anti-inflammatory and anticancer activities of **quercetin** (**Que**), which impedes human hepatocarcinoma cell line (HCCLM3 cells) propagation and invasion. Moreover, **Que** reduces levels of MMP-2 and MMP-9 indicating linking antimigratory and antiinvasive actions [91]. MMP-2 and MMP-9 levels were diminished upon treatment with **Que** for human oral cancer cells (HSC-6 and SCC-9) [92]. In the case of a cardiopulmonary resuscitation study, rats treated with 50 mg/kg **Que** for five consecutive days exhibited remarkably fewer ROS, less irritation, and less MMP-2 expression [93]. Hypertension examined using the kidneys of animals who were fed **Que** for 21 days (10 mg/kg/day) manifested a decrease in artery ROS levels and MMP-2 actions, detected by immunofluorescence and situ zymography methods [94].

**Kaempferol** (**Kae**), a bioactive molecule, has some unique features such as cardioprotective, anticancer, antidiabetic, anti-inflammatory, antiaging, and antiallergic properties [95]. It inhibited nuclear translocation of the transcription factor AP-1 to the MMP-2 promoter, decreased MMP-2 production, and prevented migration and invasion in human tongue squamous cell carcinoma cells (SCC4 cells) [96]. **Kae** is a phytoestrogen slowing the development of cancer and carcinogenesis. It reduces the production of proteins linked to metastasis, such as MMP-2 and MMP-9, in the MCF-7 breast cancer cell line [97]. 

**Naringenin** (**Nar**), extracted from multiple fruits, has anti-inflammatory and anticancer actions. Earlier research looks at how fast **Nar** changed the proliferation of lung cancer cells (A549 cells). After gelatin zymography, **Nar** lessens MMP-2 and MMP-9 levels based on its concentration [98]. Furthermore, naringenin treatment significantly reduced the nuclear translocation of NF-κB. Numerous genes, including MMP-2 and MMP-9, are modulated by the transcription factor NF-κB to control a range of cellular functions, for instance, adhesion, inflammation, and cancer metastasis [99]. **Luteoin** (**Lut**), mostly discovered in fruits, vegetables, and herbs, has anti-inflammatory and antioxidant features. The effect of **Lut** on MMP-2 and MMP-9 in the development of colon cancer was induced by azoxymethane (AOM) in BALB/c mice to investigate impacts of **Lut** on MMP-2 and MMP-9. Initially, MMP-2 and MMP-9 expression was increased, but intraperitoneal **Lut** administration (15 mg/kg) reduced the level of their expression after 21 days [100]. Recent studies both in vitro and in vivo using subcutaneous injection in nude mice (A2780 cells) have shown that the activity of MMP-2 and MMP-9 declines in ovarian cancer cells [101,102]. 

**Myricetin** (**Myr**) is abundant in vegetables, fruits, beverages, and certain therapeutic plants. Several studies found that it has antioxidant, anti-inflammatory, anticancer, and neurological functions [103]. It fights against cancer cells through several pathways, including (a) control of MMP-2 and MMP-9 activities and (b) reduction of MMP-2 protein production in colorectal cancer cells (COLO 205). Moreover, gelatin gel zymography analysis of isolated MMP-2 [104] utilizing breast cancer cells (MDA-Mb-231) indicates a strong and direct interaction between **Myr** and MMP-2 and blocks breast cancer metastasis by lowering the action of MMP-2 and MMP-9 [105].

Another study looked at how **Myr** affected the growth and spread of radioresistant lung cancer cells (A549-IR cells) via the suppression of MMP-2 and MMP-9 expression. Experimental data have shown that **Myr** prevents the growth and migration of cancer in A549 cells [106].

*Scutellaria baicalensis* Georgi (Lamiaceae) generates a bioactive **baicalein** (**Bai**) used as traditional Chinese medicine. With its antioxidant, antiviral, anti-inflammatory, antiangiogenic, and anticancer properties, **Bai** has a multitude of beneficial advantages [107]. Numerous investigations reveal that **Bai** functions as an antitumor agent via multiple mechanisms, including the regulation of MMP-2 and MMP-9 expression [108]. Further research on the anti-proliferative properties of **Bai** in melanoma cell lines (A375 and SK-MEL-28) revealed a significant reduction in MMP-2 expression in **Bai**-treated cells [109]. Moreover, **Bai** research on pancreatic neuroendocrine tumor cell line BON1 revealed a correlation between decreased MMP-2 and MMP-9 levels and a reduction in tumor invasion and migration [110]. The latest findings indicate that **Bai** contributes to the prevention of osteosarcoma metastasis. Following treatment with **Bai**, the invasive capacity of human osteosarcoma cells (CRL-1427 cells) was reduced as a consequence of low production of MMP-9 and MMP-2 [111]. **Genistein** is a flavonoid isolated from the *Leguminosae Genistin rhizome*. Its effects include antitumor, antibacterial, antioxidant, hypolipidemic, and estrogen-like properties. It may show beneficial antitumor effect by inhibiting angiogenesis and inducing tumor cell programmed death. **Genistein** had the potential to stop the human colon cancer cell line HCT116 from propagating because it is a potent MMP-9 inhibitor that prevents the growth and spread of tumors investigated in vivo tests in mice [112]. Another naturally occurring MMP inhibitor is **Silibinin** isolate from milk thistle seeds, which has antioxidant and anticarcinogenic properties. It behaves as a chemopreventive agent to stop skin cancer and influences how breast cancer metastasizes. By inhibiting the MEK/ERK cascade in a dose-sensitive way, it suppresses the production of MMP-9 in mice. **Silibinin** also inhibits cancer cell migration, and MMP-9 expression in thyroid and breast cancer cells is reduced by protecting the ECM [113,114,115]. 

The compounds that appear to be active in *Panax* root are termed **ginsenosides (GSs).** They are a class of glycosylated triterpenes, which could be referred to as saponins. Some studies highlight the diverse medicinal benefits of **GSs** on heart disease and hormonal, immunological, and neurological functions [116,117,118]. Additionally, it has been proved that **GSs** suppress the proliferation of cancers via transforming MMP-2 and MMP-9 [119,120]. Blocking the expression of these enzymes could inhibit the formation of cancerous tumors. Due to their ability to reduce MMP levels, **GSs** have the potential to be effective cancer chemopreventive medications [119,120,121,122]. Given that MMPs have a widely recognized influence on adipogenesis, **GSs** could modulate MMP activity and reduce adipogenesis in 3T3-L1 adipocytes [116].

**Amenoflavone** is a polyphenolic constituent that was found in *Selaginella tamariscina* and has been demonstrated to possess a variety of therapeutic properties [123,124]. These properties include neuroprotective, antiallergic, antioxidant, and antitumor properties. Amentoflavone suppresses NF-κB activation and modifies the expression of antimetastatic proteins, following in vivo experiments conducted on B16F-10 melanoma cells [125]. **Amentoflavone**-induced cell cycle depletion and apoptosis, which had an impact on the respiratory system in vitro in breast cancer cells, may limit the proliferation of cells [124]. The antimetastatic actions of **amentoflavone** by inhibition of MMPs, especially MMP-2 and MMP-9, have been believed to be involved in tumor progression, partly because of their ability to degrade collagen type IV, one of the major components of basement membranes [126].

**Sulforaphane** is a naturally found in large amounts in cruciferous vegetables, such as cabbage and broccoli, and has anticancer and anti-inflammatory properties [127]. It has been reported that consuming cruciferous vegetables could prevent stomach cancer. **Sulforaphane** is currently observed to initiate apoptosis and block the cell cycle of human colorectal cancer [128]. In addition, **sulforaphane** may make hepatoma cancer cells more susceptible to TRAIL-induced apoptosis through DR5 overexpression driven by ROS [129]. Recently, fundamental processes of the influence of **sulforaphane** on the nicotine-mediated stimulation of MMP-9 has been reported. It suggests **sulforaphane** impedes the nicotine-related MMP-9 activity by blocking the ROS-mediated NF-κB and MAPK (p38 MAPK, Erk1/2)/AP-1 signaling pathways that serve to treat abdominal cancer cells in humans [130].

**Caffeic acid** (**CA**), a strong and selective MMP-9 activity and transcription inhibitor, was isolated from the plant *Euonymus alatus* [133]. It is a common phenolic acid found naturally in fruits, vegetables, wine, olive oil, and coffee [131]. It has antioxidant, antiallergic, and antiproliferative functions. Caffeine has been demonstrated to have antioxidant qualities in normal cells and pro-oxidant characteristics in cancer cells [132]. Ceramide-induced NF-κB-binding capability is effectively inhibited by **CA** [133]. According to recent studies, **CA** inhibits MMP-9 enzymatic activity and gene expression, both of which are critical for colon cancer invasion and metastasis [134]. 

**Combitastatins** are derived from the willow tree *Combretum caffrum* in South Africa. Among **combitastatins**, **Combretastatin A-4** (**CA-4**) is the most popular product that can bind to tubulin MMP-2 and MMP-9 [135]. **CA-4** has antimetastatic qualities and could downregulate the production of MMPs, mostly MMP-2 and MMP-9, in A549 cells [136,137,138,139,140]. Naturally occurring plant-based compounds have recently drawn more interest due to their potential role as cancer preventatives for those who have substantial cancer risk [141,142]. The main sources of **pterostilbene** are blueberries and several grape varieties [143]. **Pterostilbene** includes similar features to resveratrol, such as antiproliferative, anti-inflammatory, anticancer, and antioxidant properties [144,145,146]. A recent study showed that **pterostilbene** inhibited the generation of MMP-2 and decreased the capacity of the cells to migrate via stimulating Erk1/2 in A7r5 cells [147]. **Decursin**, a coumarin derivative present in the roots of *Angelica gigas*, has long been utilized for remedying anemia [148]. Cell cycle death appears when it comes into contact with breast, prostate, bladder, and colon cancer cells [149,150,151]. According to recent studies, **decursin** inhibits the expression of NF-κB in breast cells and macrophages hence limiting MMP-9 generation [151,152,153].

Researchers’ interest in natural products has grown significantly in recent years because of their possible chemopreventive roles in human health and illnesses. Marine- and plant-based natural products are known to have significant antioxidant properties that fight against cellular oxidative stress and ROS, lowering the risk of diseases. Additionally, these natural products could contribute to the development of treatments for many chronic ailments including infectious diseases, cancer, diabetes, cardiovascular diseases (CVDs), neurodegenerative diseases, and inflammatory disorders [154]. In this regard, we emphasized and summarized some natural products and their mechanisms of action against a range of serious human illnesses.

### 2.4. Mechanism of MMP Regulation by Natural Products 

Over two decades, it has been demonstrated that cell inactivation could be a barrier to curing cancer [155]. It is widely known that MMPs are responsible for cell death because they induce metastasis and invasion of normal cells by degrading the ECM as well as the basement membrane [156]. The Chinese started to use the natural product **Bai,** which was extracted from the roots of *Scutelaria baicalensis* as an anticarcinogenic agent. Natural MMP regulators could deteriorate the activity and expression of MMPs in order to cure cancers after taking them in a daily diet. These active ingredients pass through the cell membrane and interact directly or indirectly with MMPs [157,158,159,160,161]. After revealing positive aspects of MMP regulation by **Bai**, other nations began to widely appreciate Bai as well as take it as a drug [156]. Although the mechanism of action relies on the type of natural products, the proposed suppression pathways of activity of natural products against MMPs are briefly described. The natural products enlisted in this review could regulate the expression and activity of MMP-2 and MMP-9 through (a) breaking hydrogen bonds of A-T or C-T of DNA and distortion of MMPs geometry around the Zn(II) center [162,163,164], (b) damaging the chromosomal sequence of DNA by generating ROS [165,166,167,168], (c) activating PKC-δ with the help of NADPH and ROS generation to activate p38-MARK [169,170,171], (d) inactivating the vascular endothelial growth factor (VEGF) [172], and (e) suppressing NF-κB signaling [173,174,175].

### 2.5. Difficulties of Natural Products Using as Medications 

Natural products have been used extensively to treat cancer. Nevertheless, their poor pharmacokinetic traits, low water solubility, low biocompatibility, limited oral bioavailability, and volatility make it difficult to employ them in clinical applications on their own [176]. Furthermore, there is little yield from spontaneous isolation of natural products [177]. Additionally, using these natural products frequently results in serious adverse consequences, such as myelosuppression, dizziness, vomiting, stomatitis, exhaustion, diarrhea, gastrointestinal pain, peripheral nerve damage, and baldness [176,177]. Despite these difficulties, natural products still have potential as medications for various diseases, including cancers as summarized above.

## 3. Conclusions and Future Prospects

This study summarized how natural products from natural fruits and vegetables as well as marine organisms have been shown to have positive impacts on cancer by downregulating the actions and/or levels of MMP-2 and MMP-9. The therapeutic effects of numerous natural products obtained from botanical and aqu atic sources in the recovery from deadly human cancers have been studied. As MMPs are implicated in the development of such disorders, a variety of natural products have been used to control MMP generation and activity. Multiple natural products have been identified as powerful inhibitors of MMP-2 and MMP-9 expression. Chemoresistance has been commonly endured by many traditional chemotherapeutics, which is the main concern and hurdle in cancer therapy along with radiation resistance. Natural products are ideal candidates for chemosensitizing cancer cells and increasing the efficacy of existing drugs. Furthermore, additional research is required to pinpoint particular regulatory systems downregulating the activity and/or upregulating the expression levels of MMP-2 and MMP-9, governed by natural products.

## Figures and Tables

**Table 1:** Marine-derived natural product downregulators for MMP-2 and MMP-9.

Product	Name	Sources	MMP-2	MMP-9	Models	Refs.
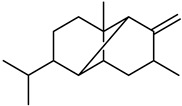	Lemnalol	*Lemnalia* sp.	Unknown	Suppression	Rats	[79]
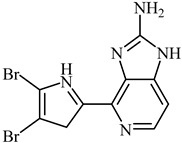	Ageladine A	*Agelas nakamurai*	Suppression	Suppression	In vitro	[80]
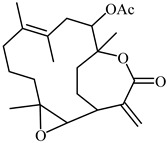	11-Episinularoide acetate	*Sinularia flexibilis*	Suppression	Suppression	HA22T cells	[81,82]
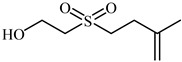	Dihydroaustrasulfone alcohol	*Cladiella australis*	Suppression	Suppression	A549 cells VSMC	[83,84]
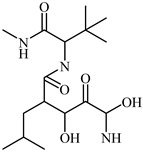	Marimastat	*Algae*	Unknown	Suppression	Mice	[85,86,87]
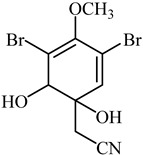	Aeroplysinin-1	*Aplysina aerophoba*	Suppression	Unknown	Endothelial cells	[88,89]

**Table 2:** Flavonoids and other compounds from fruits and vegetables, which can modulate MMP-2 and MMP-9 activity and/or levels.

Product	Name	Sources	MMP-2	MMP-9	Models	Refs.
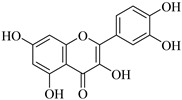	Quercetin	Vegetables, seeds, and grains	Suppression	Suppression	HSC-6 cells SCC-9 cells 2K1C rats	[91,92,93,94]
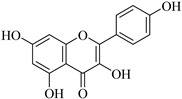	Kaempferol	Tea, cabbage, beans, tomato, strawberries, and grapes	Suppression Suppression	Unknown Suppression	SCC-4 cells MCF-7 cells	[95,96,97]
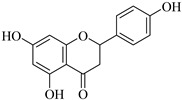	Naringenin	Grapefruit, bergamot, orange, tomatoes, and cocoa	Suppression	Suppression	A549 cells	[98,99]
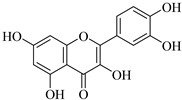	Luteoin	Salvia, broccoli, parsley, thyme, green pepper, and artichoke	Suppression Suppression Suppression	Suppression Suppression Suppression	Mice A2780 cells, mice	[100,101,102]
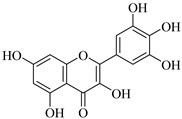	Myricetin	Tomatoes, nuts, oranges, berries, tea, and red wine	Suppression Suppression Suppression Suppression	Unknown Suppression Suppression Suppression	COLO 205 cells MDA-Mb-231 cells A549 cells	[103,104,105,106]
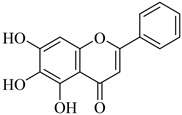	Baicalein	Root of *Scutellaria* *Baicalensis*	Suppression Suppression Suppression Suppression	Unknown Unknown Suppression Suppression	SK-MEL-28 BON1 cells CRL-1427 cells A375 cells, mice	[107,108,109,110,111]
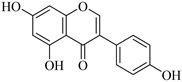	Genistein	Food, soya products	Unknown	Suppression	Mice	[112]
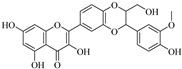	Silibinin	Fruits	Unknown	Suppression	Mice	[113,114,115]
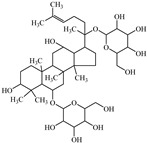	Ginsenoside	Panax root	Suppression	Suppression	3T3-L1 cells	[116,117,118,119,120,121,122]
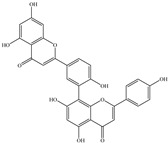	Amenoflavone	*Selaginella tamariscina*	Suppression	Suppression	Breast cancer cells B16F-10 cells	[123,124,125,126]
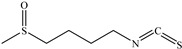	Sulforaphane	Cabbage and broccoli	Unknown	Suppression	Abdominal cancer cells	[127,128,129,130]
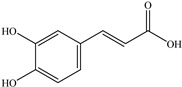	Caffeic acid	Fruits, vegetables, wine, olive oil, and coffee	Unknown	Suppression	Colon cancer cells	[131,132,133,134]
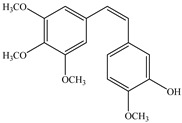	Combretastatin A-4	Willow tree	Suppression	Suppression	A549 cells	[135,136,137,138,139,140]
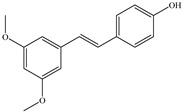	Pterostilbene	Blueberries and grape	Suppression	Unknown	A7r5 cells	[141,142,143,144,145,146,147]
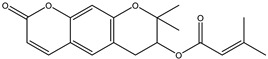	Decursin	*Angelica gigas*	Unknown	Suppression	Breast cancer cells	[148,149,150,151,152,153]
